# Adjustment to a “New Normal:” Coping Flexibility and Mental Health Issues During the COVID-19 Pandemic

**DOI:** 10.3389/fpsyt.2021.626197

**Published:** 2021-03-19

**Authors:** Cecilia Cheng, Hsin-yi Wang, Omid V. Ebrahimi

**Affiliations:** ^1^Department of Psychology, The University of Hong Kong, Hong Kong, China; ^2^Department of Psychology, The University of Oslo, Oslo, Norway; ^3^Modum Bad Psychiatric Hospital, Vikersund, Norway

**Keywords:** coronavirus disease, resilience, coping, stress, psychological well-being, adaptation, Chinese, epidemic

## Abstract

The Coronavirus Disease 2019 (COVID-19) pandemic is an unprecedented health crisis in terms of the scope of its impact on well-being. The sudden need to navigate this “new normal” has compromised the mental health of many people. Coping flexibility, defined as the astute deployment of coping strategies to meet specific situational demands, is proposed as an adaptive quality during this period of upheaval. The present study investigated the associations between coping flexibility and two common mental health problems: COVID-19 anxiety and depression. The respondents were 481 Hong Kong adults (41% men; mean age = 45.09) who took part in a population-based telephone survey conducted from April to May 2020. Self-report data were assessed with the Coping Flexibility Interview Schedule, COVID-19-Related Perception and Anxiety Scale, and Center for Epidemiological Studies Depression Scale. Slightly more than half (52%) of the sample met the criteria for probable depression. Four types of COVID-19 anxiety were identified: anxiety over personal health, others' reactions, societal health, and economic problems. The results consistently revealed coping flexibility to be inversely associated with depression and all four types of COVID-19 anxiety. More importantly, there was a significant interaction between perceived likelihood of COVID-19 infection and coping flexibility on COVID-19 anxiety over personal health. These findings shed light on the beneficial role of coping flexibility in adjusting to the “new normal” amid the COVID-19 pandemic.

## Introduction

The emergence of an atypical coronavirus, SARS-CoV-2, instigated a global outbreak of Coronavirus Disease 2019 [COVID-19; e.g., (1)]. Following identification of the earliest cases of COVID-19 in December 2019, the World Health Organization (2) declared the viral outbreak a health emergency of international concern on January 30, 2020, and then a global pandemic <2 months later. The escalating pandemic has induced anxiety and panic reactions in the general public, and the emotional responses bear some resemblance to those observed amid the severe acute respiratory syndrome (SARS) outbreak in 2003 [e.g., (3, 4)]. For instance, the panic sell-off of stocks led to a plunge in the global stock market (5), and long lines for food and the irrational stockpiling of personal protection equipment such as facemasks and hand sanitizers have been widely seen (6, 7).

Despite such resemblances, the COVID-19 pandemic is an unprecedented crisis in terms of the scope of its influence on both physical and mental health [e.g., (8, 9)]. To curb the transmission of this hitherto unknown virus, governments all over the world have enforced strict epidemic-control measures such as nationwide school closures, stay-at-home orders, and physical distancing regulations in public areas (10). Also, myriad public and private organizations have adopted teleworking policies mandating that their employees work from home (11). Although employees hold generally favorable attitudes toward home-based teleworking, the sudden drastic change in work mode left many unprepared (12). Previous research on the office-home transition has revealed major changes in the work environment to induce the most stress and anxiety in employees who feel the least prepared for this alternative work mode (13). Devastating problems arising from stressful life changes have been documented not only in adults but also in youngsters, with recent studies revealing a significant proportion of children and adolescents to have experienced psychological distress during the school-closure period (14, 15). The COVID-19 pandemic has confronted people of all ages with fundamental life changes [e.g., (16, 17)].

To grapple with the “new normal” and deal with the considerable challenges brought about by the pandemic, individuals need a considerable degree of flexibility. Psychological resilience is a widely recognized mechanism underlying the adjustment process, with coping flexibility a core component [e.g., (18)]. The theory of coping flexibility postulates that effective coping entails (a) sensitivity to the diverse situational demands embedded in an ever-changing environment and (b) variability in deploying coping strategies to meet specific demands (19). More specifically, psychological adjustment is a function of the extent to which individuals deploy problem-focused coping strategies (e.g., direct action) in controllable stressful situations and emotion-focused coping strategies (e.g., distraction) in uncontrollable ones. Inflexible coping, in contrast, has been linked to psychological symptoms. For example, individuals with heightened anxiety levels are characterized by an illusion of control [e.g., (20, 21)]. They tend to perceive all events in life as being under their control, and thus predominantly opt for problem-focused coping regardless of the situational characteristics. In contrast, individuals with depression are characterized by a sense of learned helplessness [e.g., (22, 23)]. They tend to view all events as beyond their control, and thus predominantly deploy emotion-focused coping across stressful events. Coping flexibility has been identified to foster adjustment to stressful life changes, which is indicated by a reduction in symptoms of anxiety and depression commonly experienced in stressful life transitions (24).

Applying these theories and findings to psychological adjustment during the COVID-19 pandemic, individuals higher in coping flexibility are predicted to experience lower levels of anxiety and depression than those lower in coping flexibility. Clinical trial findings on COVID-19 offer a mixture of promise and disappointment regarding the efficacy of SARS-CoV-2 vaccine candidates [e.g., (25)], and the absence of a thorough understanding of the etiology and treatment of this atypical virus has elicited widespread public panic responses. According to the theory of psychological entropy (26), uncertainty is a crucial antecedent of anxiety. In accordance with that theory, studies conducted during the pandemic have revealed unusually high prevalence rates of mental health problems such as anxiety and depression, rates ~3-fold higher than both their pre-pandemic prevalence and lifetime prevalence over the past two decades (27, 28).

In light of the transactional theory of stress and coping that highlights the importance of primary and secondary appraisals in the coping process (29), coping flexibility (secondary appraisal) is predicted to explain the association between context-specific health beliefs (primary appraisal) and mental health. Instead of perceiving the COVID-19 pandemic as aversive and uncontrollable, resilient copers tend to espouse a more complex view by recognizing both controllable and uncontrollable aspects of the pandemic. For instance, these individuals tend to take such positive actions as acquiring new information technology and digital skills to meet the demands of home-based teleworking, but engage in meditation to cope with the unpleasant emotions brought about by mandatory stay-at-home orders. Accordingly, coping flexibility is hypothesized to be inversely associated with anxiety and depression during the pandemic.

As individuals high in coping flexibility are characterized by cognitive astuteness in making distinctions in an array of stressful events (30, 31), coping flexibility is also predicted to interact with context-specific health beliefs to have a conjoint influence on mental health in the pandemic context. Although COVID-19 shares similar characteristics with other atypical coronaviruses of SARS and Middle East respiratory syndrome (MERS), the case fatality rate of COVID-19 is much lower than the others (32). Among individuals high in coping flexibility, those who tend to perceive such differences may experience lower COVID-19 anxiety than their counterparts who do not hold this perception. In this respect, mental health experienced during the pandemic is a function of both context-specific health beliefs and coping flexibility.

The present study was conducted during the “second wave” of COVID-19 infections in Hong Kong. Although the first confirmed COVID-19 case was identified on January 23, 2020, with the first death recorded 2 weeks later (33), Hong Kong remained largely unscathed by the first wave, with only sporadic cases reported and a relatively flat epidemic curve (i.e., fewer than 100 confirmed cases). However, there was a sudden surge in confirmed cases in March, when the viral outbreak swept the globe (34). The Government of the Hong Kong Special Administrative Region (HKSAR) responded to the health emergency by enacting a travel ban on non-residents, issuing compulsory quarantine orders for residents returning from overseas, and tightening various physical distancing measures in late March and early April [e.g., (35, 36)]. Special work arrangements for government employees were also implemented, and many organizations followed suit. The psychosocial impact was thus so pervasive that all sectors of society were affected. A population-based survey was therefore deemed the most appropriate method for investigating the psychological reactions to the pandemic among residents of Hong Kong. The method yields heterogeneous community samples, which maximizes representativeness and minimizes sampling errors.

## Materials and Methods

### Sample Size Determination and Power Analysis

The statistical power analysis showed that the minimum sample size was 276 in order to identify statistically significant associations among the study variables, but a larger sample size was recruited to meet the requirements for conducting principal component analysis (PCA). Considering the general rule of thumb of having at least 50 cases per factor and a maximum number of nine factors to be identified in the PCA, the pre-planned minimum sample size was 450.

### Participants and Procedures

The respondents were 481 Hong Kong adults (41% men; mean age = 45.09, *SD* = 23.42), who were recruited from a population-based telephone survey conducted by a survey research center at the first author's university. Random digit dialing was used for identifying eligible households, and then the most recent birth day method was employed to select a household member. To be eligible for participation, respondents had to be aged 18 or older, a resident of Hong Kong, able to understand Cantonese, and willing to give consent. Participation was voluntary, and all respondents who completed the survey were entered into a lucky draw for a chance to win gift certificates worth 500 Hong Kong dollars (about 65 U.S. dollars).

Trained interviewers conducted the telephone interviews using a structured questionnaire with standard questions. To foster interviewer calibration and minimize measurement bias, the survey was piloted in a small group of respondents from April 2 to 10, 2020. The final set of survey questions was amended to enhance the clarity of a few items, and then the full survey was administered from April 20 to May 19, 2020.

The study was conducted according to the ethical research standards of the American Psychological Association, and the study protocol was reviewed and approved by the human research ethics committee of the first author's university before the survey began (approval number: EA1912046 dated March 4, 2020). All respondents gave verbal consent in accordance with the Declaration of Helsinki.

### Instruments

#### Coping Flexibility

Coping flexibility was assessed by the revised Coping Flexibility Interview Schedule (37). This interview schedule was originally developed based on clinical samples (38), and was adjusted for use with heterogeneous non-clinical populations. In the pilot phase, some respondents reported difficulty in understanding the terms of primary and secondary approach coping that was currently used in our interview schedule. The interview questions were revised by combining the terms of primary and secondary approach coping into problem-focused coping and converting the term of avoidant coping style into emotion-focused coping. Problem-focused and emotion-focused coping were originally used in the transactional theory of coping (39) from which the Coping Flexibility Interview Schedule was derived. The respondents were asked to report their deployment of problem-focused (e.g., information seeking, monitoring) and emotion-focused (e.g., acceptance, relaxation) coping in controllable and uncontrollable stressful situations over the past month.

To obtain a composite score of coping flexibility indicating strategy-situation fit, the individual coping items were subsequently coded by two independent raters according to a coding scheme (40, 41) based on coping theories (39, 42). One point was given to the deployment of problem-focused coping strategies to handle controllable stressful events and/or the deployment of emotion-focused coping strategies to handle uncontrollable stressful events. Zero points were given otherwise. All of these scores were aggregated, and then averaged to obtain a composite score. Inter-rater agreement was evaluated using Krippendorff alpha coefficients (43), and the results showed no discrepancies because no subjective codings were required (Krippendorff alpha = 100%).

#### COVID-19-Related Perceptions

Both perceived likelihood and impact of COVID-19 infection were measured by a modified measure developed and validated during the SARS outbreak (44). To make this measure relevant to the present pandemic context, the context was altered from “SARS outbreak” to “COVID-19 pandemic.” Respondents gave four-point ratings to indicate their perception of the likelihood of contracting COVID-19 (1 = *very unlikely*, 4 = *very likely*) and the impact of having it (1 = *no impact at all*, 4 = *a large impact*). The measure has been found to display both criterion and predictive validity (44, 45).

#### COVID-19 Anxiety

As the events that have occurred during the COVID-19 pandemic are unprecedented, our team conducted a qualitative study in March 2020 asking participants to list all of the issues that had made them feel anxious during the pandemic. Content analysis of the results revealed 16 distinct themes regarding anxiety-provoking issues experienced amid the pandemic (see Table 1 for details). These items were compiled into a context-specific measure for assessing COVID-19 anxiety. Respondents rated each item on a scale ranging from 1 (*not worried at all*) to 4 (*very worried*).

**Table 1:** Four-factor promax-rotated factor solution for COVID-19 anxiety (*n* = 481).

**Pandemic-specific anxiety itemPers**	**Factor**			
	**Personal health**	**Others' reactions**	**Societal health**	**Economic problems**
Health of elderly people in my community	0.72			
Health of children in my community	0.72			
COVID-19 infection in my friends/social network members	0.71			
COVID-19 infection in myself and my family members	0.69			
*Contact with a COVID-19 carrier*	*0.57*	*0.50*		
Discrimination		0.80		
Quarantine stigma		0.74		
Stockpiling of basic groceries		0.68		
Stockpiling of personal protection equipment		0.53		
Government's lack of effort/ability to handle the pandemic			0.81	
Breakdown of local healthcare system			0.67	
No effective treatment for COVID-19			0.63	
Progress of my work			0.50	
Pandemic's economic implications (e.g., recession, stock market crash)				0.78
Widening of health-wealth gap in society				0.73
My financial situation				0.64
Eigenvalues	6.15	1.58	1.22	1.15
% of variance	38.41	9.87	7.60	7.22
Cronbach's alpha	0.83	0.76	0.72	0.71

*Extraction method is principal component analysis with varimax rotation with Kaiser normalization. Factor loadings below the 0.45 threshold were omitted from the table. The item with double loading (in italics) was removed from the statistical analyses*.

#### Depression

Depression was measured by the short form of the Center for Epidemiological Studies Depression Scale (46), which contains 10 items. The translated Chinese version was used in this study (47). Respondents rated each item on a four-point scale (0 = *rarely or none of the time*, 3 = *most or all of the time*). In this study, we applied the recommended cut-off score of 10 as the classification scheme [e.g., (46, 48)].

### Statistical Analysis

All statistical procedures were conducted using SPSS version 26.0 for Windows (IBM Corporation, 2019, Armonk, NY). Before hypothesis testing, PCA was performed to identify the factorial structure underlying the 16 anxiety-provoking issues. The components were rotated using the varimax method with Kaiser normalization to increase the interpretability of the findings. The number of factors extracted was determined by the Kaiser rule, with factors retained when the eigenvalue exceeded one. The total amount of variance accounted for by the factors needed to exceed 60%, a minimum criterion for factor selection widely adopted in PCA research (49). Both the Kaiser-Meyer-Olkin (KMO) measure of sampling adequacy and Bartlett's test of sphericity were first examined to check the appropriateness for analyzing the dataset, with appropriateness indicated if the KMO index was >0.50 and the test of sphericity was significant. For PCA, items with a factor loading <0.45 or double loading were removed. Cronbach alpha was used to indicate internal consistency for the items within each factor, with an alpha >0.70 considered adequate.

The potential differences among demographic groups were examined. Differences in sex were detected using an independent-samples *t*-test, and age differences using Pearson zero-order correlation analysis. In addition to testing age as a continuous variable, we also adopted a generational approach proposed by the Pew Research Center that makes comparisons across four age cohorts: (a) Millennials, who were born in 1981 or after; (b) Generation X-ers, who were born between 1965 and 1980; (c) Baby Boomers, who were born between 1946 and 1964; and (d) Silent Gen'ers, who were born before 1946 (50). A general linear model (GLM) was employed to investigate the differences among the four generations, with *post hoc* Bonferroni tests conducted if generational differences were found in any of the study variables.

Pearson zero-order correlation analysis was conducted to obtain an overview of the inter-relationships among the study variables. The hypothesized beneficial role of coping flexibility on mental health was then tested using three-step hierarchical regression analysis. First, the two demographic variables (i.e., sex and age) were entered to control for their potential effects on the criterion in question. Second, the variables of perceived likelihood of COVID-19 infection, perceived impact of COVID-19 infection, and coping flexibility were entered simultaneously. Third, the Perceived Likelihood of COVID-19 Infection × Coping Flexibility interaction and the Perceived Impact of COVID-19 Infection × Coping Flexibility interaction were entered. To address the potential multicollinearity problem, all of the variables were centered before conducting these analyses. The procedures were identical for each mental health problem included as the criterion variable. To unpack significant interaction effects, *post hoc* simple effects analysis was employed to examine the effects of COVID-19-related perception on a criterion at each level of coping flexibility.

## Results

PCA was performed because the KMO index was high (.87) and Bartlett's test of sphericity was significant (χ^2^ = 3379.31, *p* < 0.0001). The results with the principal component weights of the 16 anxiety-provoking issues are presented in Table 1. A four-factor solution was yielded, accounting for 63% of the total variance, with 38% explained by the first factor, personal health issues (e.g., “*COVID-19 infection in myself and my family members*”); 10% by the second factor, other people's undesirable reactions (e.g., “*discrimination*”); 8% by the third factor, societal health issues (e.g., “*government's lack of effort/ability to handle the pandemic*”); and 7% by the fourth factor, economic problems (e.g., “*pandemic's economic implications*”). It is noteworthy that one item (i.e., “*contact with a COVID-19 carrier*”) had a double loading with a difference of <0.10, and was thus discarded. All four factors displayed internal consistency (Cronbach alphas > 0.70), and were thus included in the subsequent analyses as indicators of COVID-19 anxiety.

The GLM results revealed a significant cross-generational difference only for anxiety over societal health, *F*_(3, 477)_ = 33.92, *p* < 0.0001, partial eta squared = 0.18. *Post hoc* Bonferroni tests indicated that Silent Gen'ers aged over 74 (*M* = 2.02, *SD* = 0.62) reported significantly less anxiety over societal health than did Millennials aged 18–39 (*M* = 2.87, *SD* = 0.66) or Generation X-ers aged 40–55 (*M* = 2.71, *SD* = 0.68), *p*s < 0.0001. However, there were no other differences regarding sex, generation, or the Sex × Generation interaction, *p*s > 0.05.

The descriptive statistics of and inter-relationships among the study variables are presented in Table 2. The average depression score was 9.85, which was very close to the cut-off score for probable depression. Adopting the standard cut-off criterion of 10, slightly more than half (52%) of the respondents were categorized as having probable depression. The probable depression group (*M* = 2.67, *SD* = 0.75) generally experienced a higher anxiety level over societal health issues than the no depression group (*M* = 2.48, *SD* = 0.73), *t* = 2.72, *p* = 0.007. In addition, the probable depression group (*M* = 0.50, *SD* = 0.21) also reported a generally lower degree of coping flexibility than the no depression group (*M* = 0.58, *SD* = 0.21), *t* (479) = −3.95, *p* < 0.0001. However, no other significant differences in depression level were found for sex or generation, *p*s > 0.21.

**Table 2:** Descriptive statistics of study variables (*n* = 481).

**Variable**	***M***	***SD***	**2**	**3**	**4**	**5**	**6**	**7**	**8**	**9**	**10**
1. Sex^a^			0.023	−0.036	0.101^*^	0.037	0.020	0.053	0.049	0.115^*^	−0.034
2. Age	45.09	23.42		−0.049	−0.035	0.092^*^	−0.089	−0.063	−0.366^**^	0.0003	−0.018
3. Likelihood of infection	2.31	0.70			0.214^**^	−0.057	0.249^**^	0.215^**^	0.226^**^	0.174^**^	0.006
4. Impact of infection	3.12	0.84				−0.156^**^	0.377^**^	0.301^**^	0.391^**^	0.275^**^	0.106^*^
5. Coping flexibility	0.54	0.21					−0.299^**^	−0.215^**^	−0.212^**^	−0.165^**^	−0.195^**^
6. Anxiety over personal health	2.57	0.76						0.546^**^	0.500^**^	0.463^**^	0.105^*^
7. Anxiety over others' reactions	2.07	0.80							0.457^**^	0.422^**^	0.116^*^
8. Anxiety over societal health	2.58	0.75								0.493^**^	0.144^**^
9. Anxiety over economic problems	2.54	0.77									0.135^**^
10. Depression	9.85	2.96									

a *Point-biserial correlation coefficients were reported instead of the typical Pearson's product-moment correlation coefficients because sex was dummy coded (0 = men, 1 = women)*.

* p < 0.05;

** *p < 0.01*.

**Figure 1:**
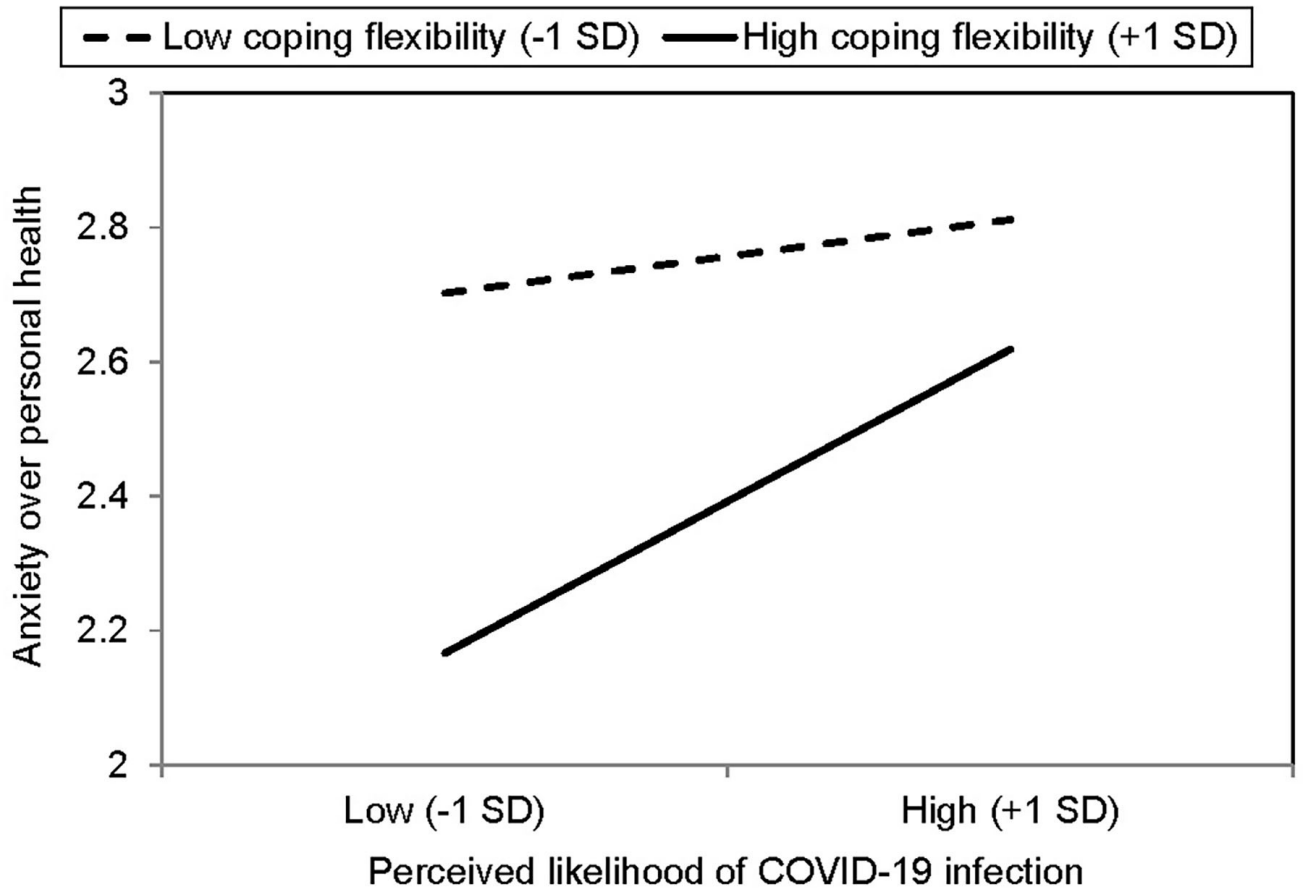
Simple effects analysis for significant interaction between perceived likelihood of COVID-19 infection and coping flexibility (*n* = 481).

Table 3 summarizes the results of hierarchical regression analysis for various mental health problems. As shown in the table, the pattern of results was highly consistent across the four types of COVID-19 anxiety; that is, all four types were positively associated with both the perceived likelihood and impact of COVID-19 infection and inversely associated with coping flexibility. There was also a significant interaction between perceived likelihood of COVID-19 infection and coping flexibility, and the results are presented in Figure 1. For individuals higher in coping flexibility, those who perceived a lower likelihood of contracting COVID-19 reported less anxiety over their own health than their counterparts who perceived a greater likelihood of such contraction. For individuals lower in coping flexibility, however, such individual differences were absent and they generally reported greater anxiety over their own health than those higher in coping flexibility. In addition, the results revealed depression to also be inversely associated with coping flexibility, although its associations with the two types of COVID-19-related perception were non-significant. In short, these findings provide support for the hypothesized beneficial role of coping flexibility in dealing with mental health issues experienced during the COVID-19 pandemic.

**Table 3:** Summary of hierarchical regression analysis by mental health problems (*n* = 481).

	**Anxiety over** **personal health**		**Anxiety over** **others' reactions**		**Anxiety over** **societal health**		**Anxiety over** **economic problems**		**Depression**	
	***B***	***SE***	***B***	***SE***	***B***	***SE***	***B***	***SE***	***B***	***SE***
Step 1	*R^2^* = 0.007		*R^2^* = 0.004		*R^2^* = 0.131		*R^2^* = 0.012		*R^2^* = 0.002	
Sex	0.033	0.070	0.074	0.075	0.084	0.065	0.174^*^	0.072	−0.210	0.277
Age	−0.003	0.001	−0.002	0.002	−0.011^**^	0.001	0.000	0.002	−0.004	0.006
Step 2	*R^2^* = 0.225		*R^2^* = 0.141		*R^2^* = 0.297		*R^2^* = 0.110		*R^2^* = 0.046	
Sex	0.007	0.063	0.053	0.070	0.048	0.059	0.154^*^	0.069	−0.236	0.274
Age	−0.001	0.001	−0.001	0.001	−0.010^**^	0.001	0.001	0.001	−0.001	0.006
Likelihood of	0.182^**^	0.045	0.176^**^	0.050	0.142^**^	0.042	0.139^**^	0.049	−0.103	0.196
infection										
Impact of infection	0.270^**^	0.038	0.224^**^	0.043	0.287^**^	0.036	0.195^**^	0.041	0.309	0.166
Coping flexibility	−0.827^**^	0.147	−0.641^**^	0.164	−0.412^**^	0.137	−0.457^**^	0.160	−2.539^**^	0.638
Step 3	*R^2^* = 0.243		*R^2^* = 0.150		*R^2^* = 0.302		*R^2^* = 0.113		*R^2^* = 0.046	
Sex	0.014	0.062	0.057	0.070	0.053	0.059	0.157^*^	0.069	−0.237	0.275
Age	−0.001	0.001	0.000	0.001	−0.010^**^	0.001	0.001	0.001	−0.001	0.006
Likelihood of	0.165^**^	0.045	0.163^**^	0.050	0.136^**^	0.042	0.131^**^	0.049	−0.099	0.198
infection										
Impact of infection	0.256^**^	0.038	0.211^**^	0.043	0.284^**^	0.036	0.189^**^	0.042	0.314	0.167
Coping flexibility	−0.826^**^	0.145	−0.642^**^	0.163	−0.410^**^	0.137	−0.457^**^	0.160	−2.539^**^	0.640
Likelihood of infection × Coping flexibility	0.571^**^	0.212	0.248	0.238	0.338	0.200	0.245	0.233	−0.056	0.932
Impact of infection × Coping flexibility	0.210	0.180	0.352	0.202	−0.091	0.170	0.095	0.209	−0.128	0.793

* p < 0.05;

** *p < 0.01*.

In addition to evaluating strategy-situation fit using composite coping flexibility scores, nuanced analysis was conducted to further examine the deployment of individual coping strategies and their associations with mental health problems. Most of the respondents (61%) reported deploying problem-focused coping to handle controllable stressful events during the pandemic, whereas just under half (45%) reported deploying that strategy to deal with uncontrollable stressful events. Fewer respondents said they had used emotion-focused coping to deal with controllable and uncontrollable stressful events (39 and 37%, respectively). Moreover, the deployment of problem-focused coping in controllable stressful events was inversely associated with anxiety over personal health and others' reactions, *p*s <0.0001, whereas the deployment of emotion-focused coping in controllable stressful events was positively associated with all four types of COVID-19 anxiety and depression, *p*s < 0.0001. However, neither problem-focused nor emotion-focused coping deployed in uncontrollable stressful events were significantly associated with any of the mental health problems, *p*s > 0.14.

## Discussion

The present study has investigated coping responses and mental health issues among the general public in Hong Kong amid the second wave of the COVID-19 pandemic. Recent studies have identified high prevalence rates of anxiety and depression among residents of COVID-19-affected regions all over the world [e.g., (28, 51)]. Our study expands this growing body of research by specifying four major factors of COVID-19 anxiety: personal health, others' reactions, societal health, and economic problems. Although the third factor is characterized primarily by societal health issues, it is interesting to note that a seemingly unrelated item “*progress of my work*” also loaded onto this factor. This perplexing finding may reflect the fact that employees' work progress has been affected more by societal factors (e.g., implementation of prevention and control disease regulations for business and premises, home-based teleworking policy) than personal factors during the pandemic.

A similar phenomenon is found for the fourth factor, economic problems. Most of the items loading onto it involved broad societal issues (e.g., economic recession, widening of health-wealth gap), but an item related to personal financial problems also did so. This finding similarly indicates that individuals' personal financial condition during the pandemic may be influenced to a great extent by the wider economy. Taken together, these interesting findings reflect the intricate interactions between the individual and society in times of crisis, thus attesting to the necessity of identifying anxiety-provoking issues specific to the pandemic in addition to assessing generic mental health issues that are context-free.

In addition to anxiety, our findings also show depression to have been prevalent among Hong Kong adults during the second wave of the pandemic, with slightly more than half the sample identified as having probable depression. Compared with respondents without depression, those with probable depression tended to experience greater anxiety related to societal health issues but not economic problems or personal health issues. These findings indicate that the unusually high prevalence of depression reported during the pandemic is largely related to health-related problems at the societal level (e.g., governmental actions to combat COVID-19, possible breakdown of local healthcare system) rather than personal health issues.

More importantly, the present study is the first to apply the theory of coping flexibility to the context of the COVID-19 pandemic, and the findings provide support for the hypothesized beneficial role of coping flexibility in relieving heightened anxiety and depression when handling the vicissitudes emerged during the pandemic. Astute strategy deployment to meet the specific demands of an ever-changing environment is essential for adjustment to the “new normal,” and a better strategy-situation fit is found to be inversely associated with both COVID-19 anxiety and depression. It is noteworthy that coping flexibility interacts with perceived susceptibility to COVID-19 infection to have a conjoint influence on COVID-19 anxiety. Even within individuals having a higher level of coping flexibility, those tend to experience fewer symptoms of COVID-19 anxiety over personal health if they display cognitive astuteness in assessing their possibility of contracting COVID-19. These novel findings provide support for the notion that the anxiety-buffering role of coping flexibility is highly context-specific (24), which is confined to infection susceptibility and anxiety over personal health in this stressful encounter. Such context-specificity is not surprising because subjective appraisals of the possibility of contracting a novel virus should be directly linked with concerns over personal health rather than other anxiety-provoking events related to non-health issues or to the society at large. Moreover, these findings further demonstrate that COVID-19 anxiety is not a unidimensional construct and should thus be studied using a multidimensional approach.

We further found the use of problem-focused coping to deal with controllable stressful events to be related to lower levels of anxiety over personal issues (i.e., personal health and others' reactions) rather than broader societal issues (i.e., societal health, economic problems). It is also noteworthy that the use of emotion-focused coping to handle controllable rather than uncontrollable stressful events was related to higher COVID-19 anxiety and depression, a finding consistent with previous studies on clinical samples of depression (22). Although the unprecedented COVID-19 pandemic is objectively an uncontrollable stressor due to its uncertain nature, the theory of coping flexibility highlights the importance of identifying aspects of life that are controllable and distinguishing these aspects from most other uncontrollable ones in a stressful encounter. For example, when a person high in coping flexibility fails to buy facemasks after visiting many stores, this person still regards the problem as controllable and keeps trying a variety of alternative means (e.g., placing orders in overseas online stores, seeking advice from members of WhatsApp groups). It is the cognitive astuteness in distinguishing between controllable and uncontrollable life aspects that fosters adjustment to stressful life changes.

Such situational differences in coping effectiveness indicate that neither problem-focused nor emotion-focused coping is inherently adaptive or maladaptive. The role of effective coping in mitigating mental health problems depends largely on the extent to which a deployed strategy meets the specific demands of the stressful encounter concerned. For instance, playing online games or browsing social network sites can be stress-relieving during leisure time (52, 53), but prolonged gameplay or social media use can impair work or academic performance while working or studying from home (54). These findings are in line with the theory of coping flexibility, highlighting the beneficial role of flexible coping in soothing mental health problems experienced during the pandemic.

The present findings also have practical implications. Given the beneficial role of coping flexibility, clinicians may work with clients to enhance coping effectiveness with regard to strategy-situation fit. Stress management intervention may involve sharpening clients' skills for (a) distinguishing the key demands stemming from an array of stressful events; (b) assessing whether or not such demands are amendable to a change in effort (i.e., controllable or uncontrollable); (c) applying the meta-cognitive skill of reflection to evaluate strategies that best match the specific demands of diverse stressful situations; and (d) subsequently deploying the most appropriate strategy to handle each stressor. Such flexible coping skills are especially useful for dealing with the psychological distress elicited by a pandemic involving an assortment of stressful events.

Coping flexibility may also be valuable at a broader level because the unpredictable progression of the COVID-19 pandemic across successive waves presents varying challenges for public health authorities worldwide. For instance, the shortage of personal protection equipment aroused immense public anxiety in Hong Kong during the first wave owing to the sudden surge in demand for facemasks and hand sanitizer. After the supply of such equipment had been stabilized, however, new societal problems emerged. For example, during the second wave, public commitment to observing physical distancing measures began to wane owing to “pandemic fatigue” (55). Public health authorities may need to adopt a certain degree of flexibility in monitoring and identifying emerging issues to allow the timely adjustment of extant disease-control measures or the formulation of new ones to mitigate changing public health threats.

Despite its important findings, several study limitations must be noted. The survey was conducted during the second wave of the pandemic, when the epidemic curve climbed to a high level and then leveled off for a few months before reaching a further peak in the third wave in July and August, 2020 (34). As the COVID-19 pandemic continues to evolve in an unpredictable manner, some of the anxiety-provoking issues identified in this study may no longer elicit anxiety to the same extent in future waves. The list of issues eliciting COVID-19 anxiety should thus be updated in future research. Given the time sensitivity of these issues, pilot testing is essential to evaluate their relevance in particular phases of the pandemic.

Further, although our findings offer robust support for the hypothesized beneficial role of coping flexibility amid the pandemic, previous meta-analysis indicated that that beneficial role is more prominent in collectivist than individualist regions (19). A fruitful direction for future research would thus be to replicate the present design in individualist countries, allowing cross-cultural comparisons to be made. In addition to cultural differences, there may also be considerable variations among Chinese adults residing in different regions, as the epidemic trajectory has varied greatly among cities in the Greater Bay Area, such as Guangzhou and Macau (56). Greater effort can be made to compare the prevalence of psychological disorders and coping processes among Chinese residents of diverse regions.

## Data Availability Statement

The raw data supporting the conclusions of this article will be made available by the authors, without undue reservation.

## Ethics Statement

The studies involving human participants were reviewed and approved by the study protocol was reviewed and approved by the Human Research Ethics Committee of the University of Hong Kong (approval number: EA1912046 dated March 4, 2020). Written informed consent for participation was not required for this study in accordance with the national legislation and the institutional requirements.

## Author Contributions

CC contributed to project design and administration, coordinated the data collection, performed the statistical analysis, and wrote the first draft of the manuscript. H-yW contributed to project design, survey creation, statistical analysis, and data interpretation. OE contributed to data interpretation and writing parts of the manuscript. All authors contributed to the article and approved the submitted version.

## Conflict of Interest

The authors declare that the research was conducted in the absence of any commercial or financial relationships that could be construed as a potential conflict of interest.
